# Radiological risks in Nasser lake water and their health and environmental implications

**DOI:** 10.1038/s41598-025-95257-7

**Published:** 2025-04-25

**Authors:** Khaled Ali, Ahmed Abu-Taleb, Abd El-Baset Abbady, Shaban Harb

**Affiliations:** Physics Department, Faculty of Science, South Valley University, Qena, Egypt

**Keywords:** Radiological activity, Annual effective dose, Cancer risk, Mortality risk, Morbidity risk, Health risk assessment, Environmental sciences, Natural hazards, Physics

## Abstract

This study aimed to evaluate the activity concentrations of naturally occurring radionuclides (NORs) in Nasser Lake water, assess the associated radiological risks, and investigate the potential health and environmental impacts. The presence of these NORs is attributed to both natural geological formations, such as uranium-rich granitic and metamorphic rocks, and anthropogenic activities, including agricultural runoff. Water samples were analyzed for radium-226 (Ra-226), thorium-232 (Th-232), and potassium-40 (K-40). The Ra-226 concentration ranged from 0.08 ± 0.003 to 1.28 ± 0.06 becquerel per liter (Bq/l), mostly between 0.2 and 1.0 Bq/l, reflecting geological and anthropogenic influences. The symbol (±) represents the measurement uncertainty associated with gamma spectrometric analysis. According to international radiation safety guidelines, Ra-226 levels below 1 Bq/l are considered safe for consumption. The Th-232 concentration varied from 0.04 ± 0.001 to 0.96 ± 0.06 Bq/l, showing significant spatial variation. Similarly, K-40 concentrations ranged from 1.35 ± 0.11 to 16.57 ± 1.43 Bq/l, with some notably high values. The annual effective dose (E_*ff*_) ranged from 15.8 to 266.15 micro sievert per year (µSv/y) for adults, reaching 362.92 µSv/y for children and 221.54 µSv/y for infants. The doses for children and infants exceeded the recommended thresholds. Cancer risk (CR) assessments showed that men’s mortality risks ranged from 2.56 × 10^−5^ to 4.10 × 10^−4^, while women’s ranged from 2.68 × 10^−5^ to 4.29 × 10^−4^. Morbidity risks varied between 3.72 × 10^−5^ and 5.95 × 10^−4^ for men and 3.89 × 10^−5^ to 6.22 × 10^−4^ for women. These risks correlate with specific lake locations, highlighting hot spots with elevated radioactive content. Water acidity levels (pH) ranged from 6.23 to 7.9, indicating predominantly neutral to slightly alkaline conditions. These variations correlated with electrical conductivity (EC), reflecting complex interaction between pH, EC, and NORs. The study assesses gamma radiation hazards from external exposure and internal risks from alpha-emitting radionuclides, such as Ra-226 and Th-232, through water ingestion. While most samples comply with standard radiation limits, elevated radionuclide levels in certain areas pose potential health risks, particularly for vulnerable populations like children. Continuous monitoring of radiological parameters in Naser Lake is essential to trace long-term trends and ensure safety compliance. Additionally, advanced water treatment methods could help mitigate radionuclide concentrations in affected areas.

**Supplementary Information:**

The online version contains supplementary material available at 10.1038/s41598-025-95257-7.

## Introduction

Nasser Lake was formed following the construction of the High Dam in Aswan, Egypt, during the 1960s. As one of the world’s largest artificial lakes, it spans approximately 5250 km^2^ with a total storage capacity of 162 billion cubic meters of water (BCM). Additionally, Nasser Lake plays a crucial role in Egypt’s food security, serving as both a major water reservoir and a vital ecological zone. It sustains a diverse aquatic ecosystem, providing a habitat for numerous fish species, which supports local fisheries and contributes to the livelihoods of fishing-dependent communities. The geological composition surrounding Nasser Lake is diverse, featuring granitic, metamorphic, and gneissic formations in the southern region, while the northern region is characterized by Nubian sandstones and basaltic formations^1^. The lake’s sediments primarily consist of silt and sand, derived from volcanic and sedimentary formations, as well as from underlying and adjacent geological structures. These geological characteristics play a crucial role in the transport and accumulation of naturally occurring radionuclides (NORs), including radium-226 (Ra-226), thorium-232 (Th-232), and potassium-40 (K-40)^2,3^. While K-40 poses a relatively lower radiological risk, Ra-226 and Th-232 can accumulate in sediments, potentially degrading water quality and increasing exposure risks for aquatic organisms and human populations^4,5^. The presence of NORs in Nasser Lake is attributed to both natural and anthropogenic sources, including both natural processes (erosion and weathering) and human activities. Erosion and weathering of uranium- and thorium-rich rocks contribute to the gradual transport of these NORs into the lake through surface runoff and groundwater seepage. Additionally, the region’s Precambrian formations and tectonic fault lines facilitate the movement of these elements into the lake’s water system^6,7^. Conversely, human activities such as mineral mining, industrial discharges, and agricultural runoff may contribute to elevated NORs levels in certain areas. Although natural erosion remains the primary source, further research is needed to quantify the specific contributions of anthropogenic activities to radionuclide contamination^8,9^. A comparison with historical data reveals variations in radiological activity over time. These fluctuations underscore the dynamic nature of radionuclide transport and accumulation, driven by geological changes, hydrological processes, and human activities^10,11^. To better understand these variations and their potential implications, long-term monitoring studies are essential for tracking radiological contamination trends over time. It is important to note that comparisons with international guidelines, such as those set by the World Health Organization (WHO)^12^, are based on reference levels that serve as advisory thresholds rather than strict regulatory limits. Although numerous global studies have explored radiological contamination in aquatic environments, research on Egyptian lakes—especially Nasser Lake—remains scarce, with only limited assessments of its radiological risks. This study bridges this research gap by conducting a detailed evaluation of natural radioactivity in the lake’s waters using advanced gamma spectroscopy techniques. This research aims to: (1) measure the activity concentrations of NORs in the water of Nasser Lake, (2) establish a baseline for radiological activity to support future comparative studies, (3) develop spatial distribution maps to enhance understanding of radionuclide dispersion patterns, and (4) assess potential gamma radiation hazards and their implications for human health and the aquatic ecosystem. Given Nasser Lake’s vital role in Egypt’s water supply and ecological balance, a thorough understanding of its radiological status is crucial for ensuring safe water usage and ecosystem sustainability. Furthermore, implementing mitigation strategies—such as advanced water treatment technologies and regulatory measures—is crucial for preserving water quality and reducing long-term health risks^13^. By offering a comprehensive analysis of radiological risks and contamination trends, this study contributes to environmental risk management and enhances public health awareness in the region.

## Experimental procedure

### Sample collection and preparation

A total of 150 water samples were systematically collected from various locations around Nasser Lake at the end of the spring season, ensuring representation of the lake’s diverse geographical and geological characteristics. The samples were collected in clean, pre-labeled Marinelli beakers (1-liter capacity), sealed in airtight polyethylene containers wrapped with parafilm to prevent any external contamination or loss of radon gas and transported to the laboratory under controlled conditions to maintain sample integrity before analysis^14^. To ensure reliable results, the samples were stored for over more one month to ensure secular equilibrium between Ra-226, Th-232, and their respective decay progeny, allowing for accurate gamma spectrometric analysis. Given the relatively short half-lives of Ra-228 (5.75 years) and Th-228 (1.91 years), the storage period was sufficient to achieve equilibrium between Th-232 and its short-lived decay products, ensuring accurate measurement of radionuclide activity concentrations. Each sample was measured continuously for 24 h to ensure accurate data acquisition. Sample preparation followed standard gamma spectroscopy protocols, including filtration to remove particulate matter that could interfere with the analysis^15^. Acidity (pH) and electrical conductivity (EC) were measured on-site using portable instruments^16^. All measurements were conducted following standardized calibration procedures to enhance accuracy and minimize uncertainty.

### Analytical methodology

#### Measurement methods for radionuclide activity concentrations

Gamma-ray spectrometry is an effective technique for detecting and quantifying both natural and anthropogenic radioactive materials. In this study, gamma-ray spectrometry was employed to measure the activity concentrations of NORs in the collected water samples^5^. The detection mechanism relies on the interaction of gamma photons with matter, primarily through photoelectric absorption, Compton scattering, and pair production transferring photon energy to electrons and generating measurable signals. During these interactions, primary electrons lose energy via ionization and excitation, leading to the formation of electron-hole pairs or secondary charged particles, depending on the detector material. For this study, a thallium-doped sodium iodide scintillation detector (NaI(Tl)) was used. The NaI(Tl) detector follows standardized gamma spectrometry protocols, ensuring accurate detection of a broad range of radionuclides. The system consists of a 3 × 3-inch NaI(Tl) detector (S-1212-I model) coupled with a 1024-microcomputer multichannel analyzer (5510 Ortec Norland). Its energy resolution is 7.5% at 662 keV, with a peak gamma-ray efficiency of 2.3 × 10^−2^ at 1332 keV and an operational bias voltage of 805 V DC. To minimize background radiation, the detector is housed inside a massive cylindrical lead shield with a thickness of 50 cm^5^. This configuration allows for reliable identification and quantification of multiple radionuclides, including Ra-226, Th-232, and K-40, in compliance with established measurement standards. The absorbed gamma energy within the NaI crystal is re-emitted as light, with thallium activation enhancing the light yield and overall detection efficiency. This activation ensures improved sensitivity, enabling more precise measurements of gamma radiation^17^. To determine the activity concentrations of NORs in the water samples, the gamma-ray spectra were analyzed. The activity concentration (A) in Bq/l of a given radionuclide was calculated using the following equation^5^:

$$\:A=\frac{Count\:Rate}{P\times\:V\times\:\epsilon\:}$$

where the C*ount Rate* (counts per second, cps) is measured in the photopeak for each energy line (corrected for background counts), *P* is the emission probability (unitless), *V* is the sample volume (in liters), and ε is the detection efficiency (unitless). The activity concentrations of the NORs were determined based on their respective gamma photo-peaks. In the case of Th-232 and Ra-226, their decay products—such as Pb-212, Pb-214, Bi-214, Ac-228, and Tl-208—were used to confirm secular equilibrium, as the half-lives of these daughter radionuclides are significantly shorter than those of their parent isotopes^5^. Gamma spectroscopy provides insight into natural radiation levels in aquatic environments and helps evaluate potential environmental and health risks. Among NORs in surface and groundwater, Ra-226 activity was determined using the NaI(Tl) scintillation detector by analyzing the spectra of its decay products, notably at 295, 352, 609, 1120, and 1765 keV. Similarly, Th-232 activity was evaluated by analyzing the spectra of its decay products at 238, 911, and 2614 keV through the detection of their characteristic emissions. K-40 activity was assessed by detecting its characteristic gamma emission at 1460 keV^5^.

#### Calculation of annual effective dose

The total effective annual dose (E_*ff*_) quantifies the radiation dose absorbed by humans each year due to gamma-emitting radionuclides, such as Ra-226 and Th-232, present in drinking water. E_*ff*_ is a crucial parameter for evaluating potential health risks associated with long-term radiation exposure through drinking water, especially since radiation susceptibility varies by age group. The E_*ff*_ is calculated using the following equations^5,18^ :

$$\:{E}_{ff}=K\times\:G\times\:A$$

where G is the annual water consumption rate (liters/year), and K is the dose conversion factor (Sv/Bq), which refers to the committed effective dose per unit intake for each radionuclide type and age category^19,20^. Consumption rates and dose conversion factors vary by age group (adults, children, and infants). The International Commission on Radiological Protection (ICRP)^21^ reports that adults consume 500 L, children 350 L, and infants 150 L of water annually. These differences in consumption rates significantly influence the radiation doses to which individuals are exposed, as the total dose depends on both the volume of water consumed and its radionuclide concentration. Ra-226 has dose conversion factors of 2.8 × 10^−7^ Sv/Bq for adults (> 17 years), 6.2 × 10^−7^ Sv/Bq for children (2–7 years), and 9.6 × 10^−7^ Sv/Bq for infants (1–2 years)^22^. Th-232 has dose conversion factors of 2.3 × 10^−7^ Sv/Bq for adults, 3.5 × 10^−7^ Sv/Bq for children, and 4.5 × 10^−7^ Sv/Bq for infants^22^. Higher dose conversion factors for children and infants reflect their greater radiation sensitivity due to smaller body sizes and higher cell division rates. This underscores the necessity of stringent monitoring of radionuclide concentrations in drinking water, particularly for vulnerable age groups. Although K-40 is a naturally occurring radionuclide, its contribution to radiation exposure through drinking water is minimal due to its unique biological regulation. Unlike Ra-226 and Th-232, K-40 is primarily absorbed through food, and its levels in the body are controlled by homeostatic mechanisms. Additionally, its dose conversion factor is significantly lower: 6.2 × 10^− 9^ Sv/Bq for adults, 2.1 × 10^− 8^ Sv/Bq for children, and 4.2 × 10^− 8^ Sv/Bq for infants, making its impact on total dose negligible compared to other radionuclides^22^. The calculated E_*ff*_ values for different age groups were compared with international reference limits established by health and safety organizations, such as the WHO^12^ and the IAEA^7^. This comparison helps determine whether the radiation levels in drinking water remain within safe limits for human consumption. Ensuring that E_*ff*_ remains below these reference thresholds is essential for minimizing long-term health risks and guiding necessary safety measures. Continuous monitoring and regulation of radionuclide concentrations in water sources is essential to safeguard public health^13^.

#### Assessment of long-term health risks: cancer risk

The cancer risk (CR) represents the probability of developing cancer due to long-term ingestion of NORs through drinking water. In this study, the assessment primarily focuses on Ra-226, a primary contributor to the total radiation dose from ingested water. CR is calculated using the following equation^23^:

$$\:CR={A}_{Ra}\times\:RC\times\:WR\times\:LE$$

where A_Ra_ represents the activity concentration of Ra-226 in Bq/l. RC is the risk coefficient (7.17 × 10^−9^ Bq^−1^ for CR mortality and 1.04 × 10^−8^ Bq^−1^ for CR morbidity). These coefficients reflect age-related susceptibility to radiation-induced cancer, ensuring a comprehensive risk assessment. WR is the annual water consumption rate, calculated as: $\:\text{W}\text{R}=\:2\hspace{0.17em}\text{L}/\text{d}\text{a}\text{y}\times\:365\hspace{0.17em}\text{d}\text{a}\text{y}\text{s}/\text{y}\text{e}\text{a}\text{r}=730$^24^, and LE is life expectancy in years. For this study, life expectancy values of 61.2 years for males and 64.1 years for females were used^12^. This model provides separate estimates for CR mortality and morbidity for both genders, accounting for differences in life expectancy and susceptibility to radiation exposure. These results help evaluate the long-term health risks associated with NORs ingestion through drinking water. Given the potential radiological impact, these findings underscore the critical need for strict regulation and monitoring of radinuclide concenterations in drinking water to mitigate long-term cancer risks.

## Results and discussion

Analyzing the radiological properties of drinking water is crucial for understanding the health and environmental risks of NORs. This section presents the study’s findings, evaluating radioactive elements, environmental indicators, risk indices, and associated health risks. The study systematically discusses these factors, highlighting both the immediate and long-term impacts of radioactive elements in water. It begins with the fundamental radioactive activity, including measurements of NORs. These measurements, obtained through gamma spectrometry analysis, serve as essential inputs for subsequent calculations. The study analyzes E_*ff*_, assessing radiation exposure across age groups: adults, children, and infants. The results are compared with internationally accepted reference values to evaluate potential risks associated with water consumption. The study also discusses long-term health risks, including the estimated CR for men and women, based on mortality and morbidity probability values. This provides a comprehensive overview of the long-term health effects of cumulative radiation exposure. Additionally, the study measured the physical and chemical properties of water, including pH and EC. The pH level indicates whether the water is acidic or alkaline, with the ideal range for drinking water being 6.5–8.5. EC reflects the concentration of dissolved salts in water and serves as a key indicator of water quality^13^. These physiochemical values complement the radiological findings, providing a more comprehensive assessment of the water’s overall quality and suitability for consumption. The key findings, including the maximum and minimum values of radionuclide activity, E_*ff*_, CR, and water properties, are summarized in Table 1. Detailed measurements for all samples are available in the supplementary materials (Table S1).

**Table 1:** Summary of maximum, minimum, and average radionuclide activity concentration, water properties, annual effective dose, and cancer risk in Nasser lake samples.

Parameter	Minimum	Maximum	Mean
Radionuclide activity concentration (Bq/l)			
Ra-226	0.08 ± 0.003	1.28 ± 0.06	0.59 ± 0.03
Th-232	0.04 ± 0.01	0.96 ± 0.06	0.36 ± 0.05
K-40	1.35 ± 0.11	16.57 ± 1.43	8.6 ± 0.75
Water properties			
ph	6.23	7.9	6.99
EC (µs/cm)	569	1863	998.43
Annual effective dose (µSv/y)			
Adults	15.8	266.15	123.7
Childs	22.26	362.92	171.9
Infants	13.74	221.54	105.6
Cancer risk (mortality)			
Man	0.257	4.1	1.88
Women	0.27	4.29	1.98
Cancer risk (morbidity)			
Man	0.37	5.95	2.74
Women	0.39	6.23	2.87

### Activity concentrations of natural radionuclides in water samples

**Fig. 1:**
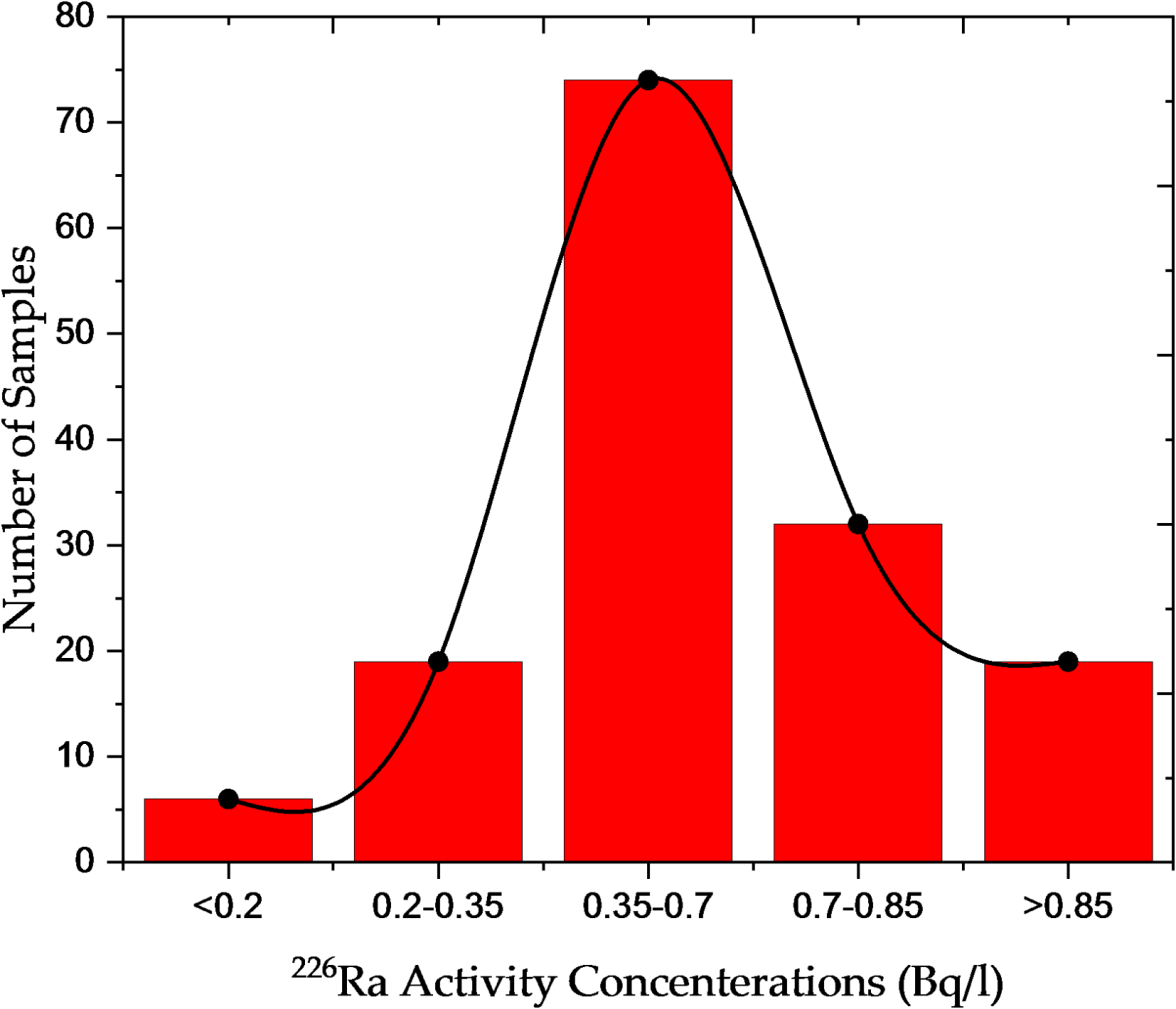
: Distribution of Ra-226 activity concentrations in Nasser Lake water samples.

**Fig. 2:**
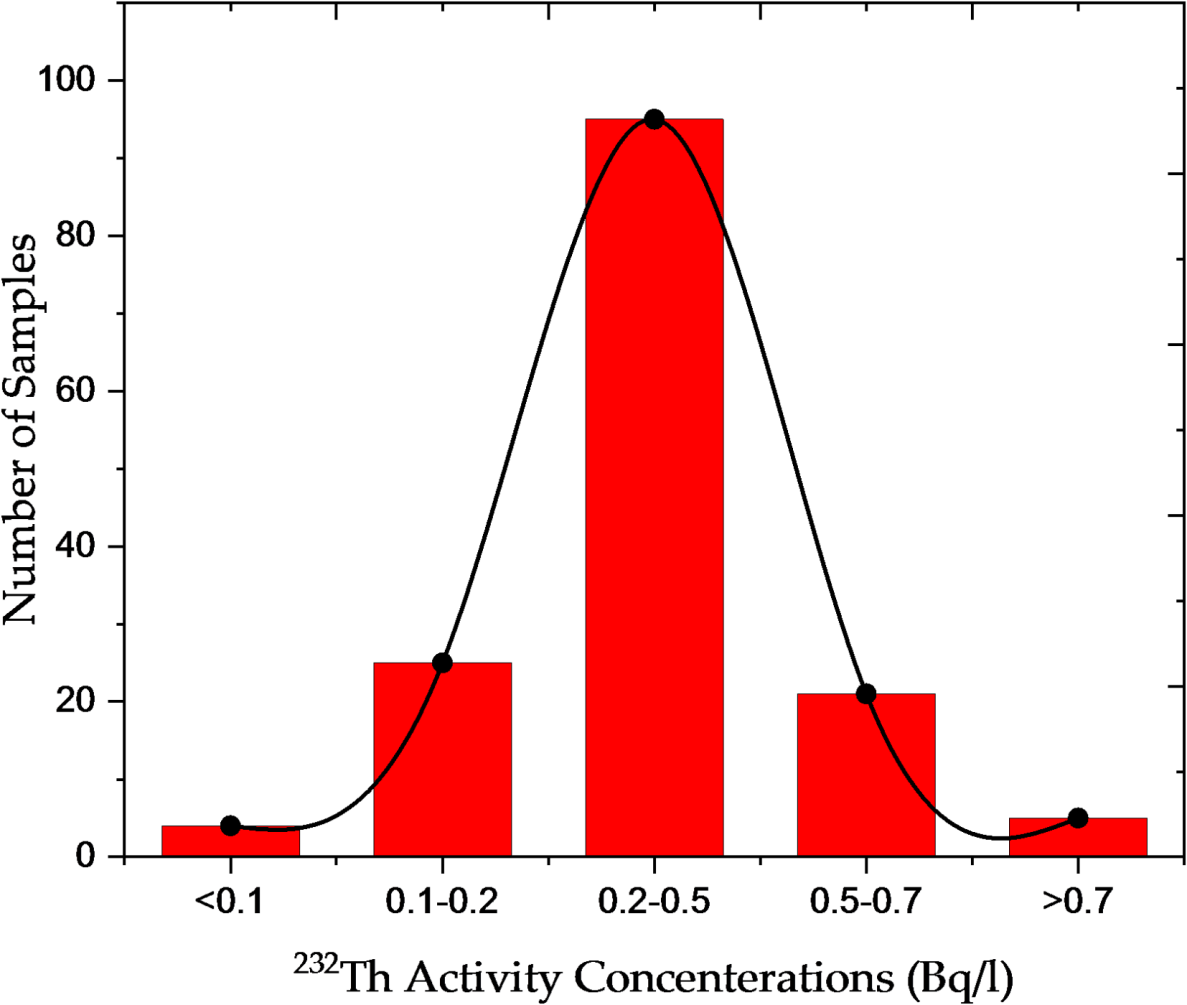
Distribution of Th-232 *activity* concentrations in Nasser Lake water samples.

**Fig. 3:**
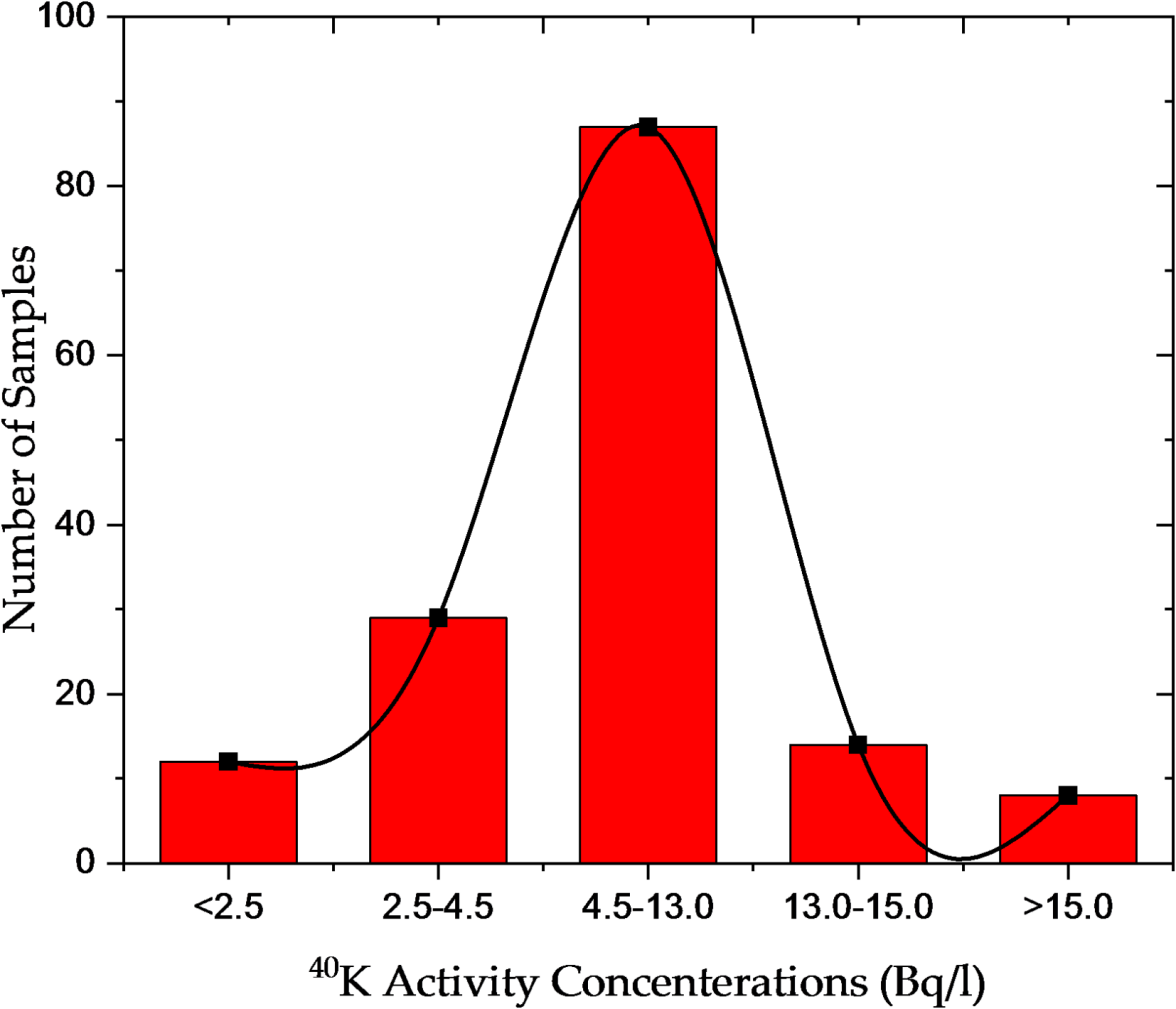
Distribution of K-40 activity concentrations in Nasser Lake water samples.

This section presents the activity concentrations of NORs in Nasser Lake water, measured using gamma spectroscopy. Figures 1, 2 and 3 illustrate the distribution of Ra-226, Th-232, and K-40 concentrations in the analyzed water samples. A frequency distribution approach was adopted to clearly represent the spatial variability of radionuclide concentrations across the lake. The concentration range for each radionuclide was divided into predefined intervals based on the observed distribution patterns of the measured values, facilitating data interpretation. The horizontal axis represents these predefined intervals, which were selected according to the observed distribution patterns in different samples, while the vertical axis indicates the number of locations where concentrations fall within each range. This approach offers a clearer understanding of how radionuclide activity levels vary across different areas of the lake, making it easier to interpret radiological variations between locations and enhancing the scientific accuracy of the analysis. The concentrations of Ra-226 ranged from 0.08 ± 0.003 Bq/l (sample W122) to 1.28 ± 0.06 Bq/l (sample W99). In this context, the symbol (±) represents the measurement uncertainty (statistical error), where the number following ± indicates the measurement uncertainty (typically the standard deviation), reflecting the precision of gamma spectrometry measurements. These variations are primarily influenced by local geological formations, particularly uranium-rich granitic and metamorphic rocks in the southern region of the lake, as well as Nubian sandstones in the north. Additionally, interactions with groundwater and sediment characteristics contribute to the distribution of radionuclides. Most samples exhibited moderate concentrations within the range of 0.2–1.0 Bq/l, based on international guidelines which consider Ra-226 levels below 1 Bq/l as within acceptable limits for drinking water safety, as recommended by the WHO^12^ and IAEA^22^. However, a few samples recorded higher values, indicating localized variations in radionuclide accumulation (Fig. 1). Ra-226, a naturally occurring radionuclide present in trace amounts in the earth’s crust, tends to accumulate in water due to geological factors, particularly in areas with uranium-rich deposits. Elevated Ra-226 levels in drinking water pose significant long-term health risks, including an evaluated risk of bone cancer and other radiation-induced health effects resulting from prolonged exposure.

The concentrations of Th-232 ranged from 0.04 ± 0.001 Bq/l (sample W122) to 0.96 ± 0.06 Bq/l (sample W120). Th-232, a naturally occurring radionuclide, is generally present at lower concentrations than Ra-226. Noticeable variations were observed, with higher concentrations detected in samples W107, W116, and W120 (Fig. 2). Unlike Ra-226, Th-232 is less soluble in water, which influences its distribution in aquatic environments. Although the health risks of Th-232 are less studied compared to Ra-226, prolonged exposure to elevated concentrations—particularly through inhalation of radioactive particles—may increase the risk of lung canser and damage to internal organs.

The concentrations of K-40 ranged from 1.35 ± 0.11 Bq/l (sample W12) to 16.57 ± 1.43 Bq/l (sample W85). K-40, a naturally occurring isotope of potassium, is one pf the primary sources of natural radiation exposure. Its concentrations showed moderate variability, with higher levels detected in samples W47, W85, and W149 (Fig. 3). Although K-40 is an essential element for the human body and contributes to natural background radiation, its presence in water is generally considered to pose lower health risks compared to Ra-226 and Th-232 due to its biological role and lower radiotoxocity. The data indicate that Ra-226 and Th-232 exhibit greater variability in concentration, with Ra-226 exhibiting the highest degree of variability. Unlike K-40, which is widely distributed in nature, the concentrations of Ra-226 and Th-232 are more strongly influenced by local geological formations and hydrogeological conditions of water sources. Higher concentrations of these NORs suggest an increased risk of radiological exposure, emphasizing the need for continuous monitoring of water quality.

Table 2 compares the activity concentrations of NORs from this study with those reported in other global regions. In Egypt, studies in the  1990s reported an average Ra-226 concentration of 0.4 Bq/L, whereas our findings indicate a range of 0.08 to 1.28 Bq/l. This may indicate a gradual increase in certain areas, potentially driven by geological leaching and anthropogenic influences. The results reveal significant variability in radionuclide concentrations, influenced by factors such as local geology, hydrology, and environmental conditions. Ra-226 concentrations in this study fall within a moderate range, higher than in some previous studies but lower than others, reflecting regional variations in uranium-rich minerals deposits and leaching processes. Th-232 concentrations align with global trends but exhibit variability due to the differences in thorium solubility and localized geological conditions. In contrast, K-40 levels were generally lower compared to global averages, likely reflecting regional variations in potassium-rich geological formations. These findings emphasize the role of both natural and human-induced factors in determining radionuclide levels in water. They highlight the necessity for localized radiological assessments to ensure water safety and minimize exposure risks.

**Table 2:** Comparison of radionuclide activity concentrations with the other local studies.

Area	Activity concentrations (Bq/l)			References
	Ra-226	Th-232	K-40	
Naser Lake	0.08 ± 0.003–1.28 ± 0.06 (Mean: 0.59 ± 0.03)	0.04 ± 0.01–0.96 ± 0.06 (Mean: 0.36 ± 0.05)	1.35 ± 0.11–16.57 ± 1.43 (8.6 ± 0.75)	Present work
Qena	Mean: 0.54 ± 0.03	0.40 ± 0.03	5.10 ± 0.44	^5^
Assiut	Mean: 0.203	Mean: 0.081	Mean: 0.688	^24^
Gebl Elba	1.6–11.1	0.21–0.97	9.7–23	^25^
Qena	Mean: 0.08	Mean: 0.04	–	^26^
Safaga-Quseir	Mean: 0.1	Mean: 0.05	–	

### Impact of annual effective dose on different age groups

**Fig. 4:**
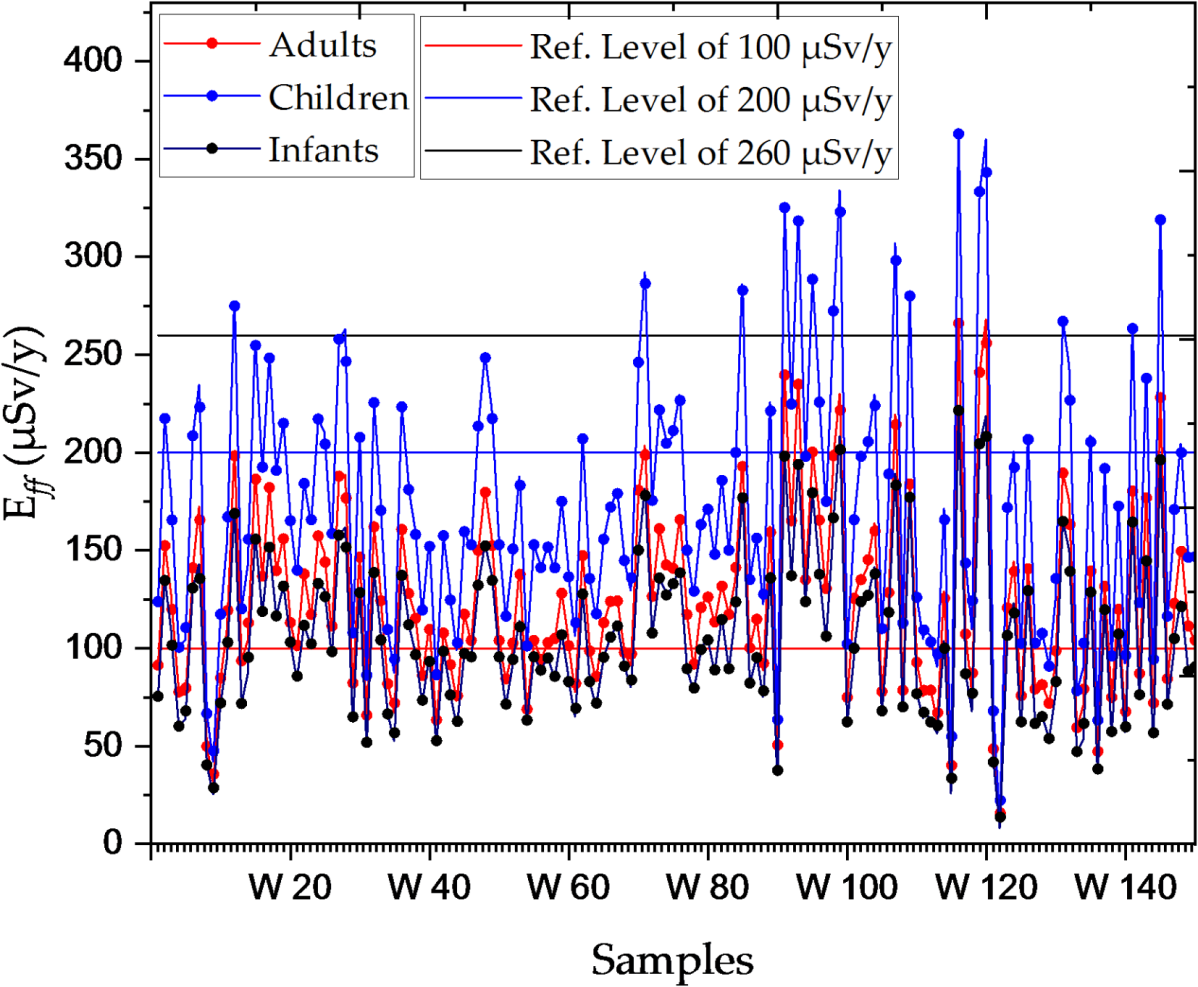
Comparison of annual effective dose across different age groups in Nasser Lake.

The E_*ff*_ for adults in most water samples falls below international reference values. For instance, sample W1 (91.4 micro sievert per year (µSv/y)) remains within the safe limit of 100 µSv/y^27^; however, higher doses in samples such as W116 (266.15 µSv/y) and W119 (241 µSv/y) exceed this threshold, suggesting localized radiation hotspots in Nasser Lake. Although these elevated doses are not immediately hazardous, prolonged exposure could lead to long-term health effects. For children, most samples indicate higher radiation doses compared to adults. Some, such as W91 (325.19 µSv/y) and W116 (362.915 µSv/y), exceeded the safe limit of 200 µSv/y^27^. Although these levels do not pose an immediate threat, children’s increased sensitivity to radiation necessitates continuous monitoring to mitigate potential long-term health risks. Infants generally receive lower doses than adults and children; however, certain samples—such as W116 (221.54 µSv/y) and W119 (204.54 µSv/y)—suggest potential long-term risks with continued exposure. The safe limit for infants is 260 µSv/y^27^, and while most samples remain within safe limits, areas with elevated values require ongoing surveillance to prevent potential health hazards. Regular assessments and continuous monitoring are crucial to ensure radiation levels in Nasser Lake remain within safe limits, particularly for vulnerable groups such as children, and infants. Comparable studies on radon exposure in drinking water, such as research conducted at Giresun University, reported E_*ff*_ mean ranging from 9.9 to 150.4 µSv/y due to ingestion^28^. Such comparisons provide valuable context for evaluating current radiological risks associated with water sources. Figure 4 illustrates the variation in annual effective dose across different age groups, highlighting the elevated exposure levels observed in children and certain localized hotspots.

### Cancer risk analysis due to radioactive activity in Nasser lake water

**Fig. 5:**
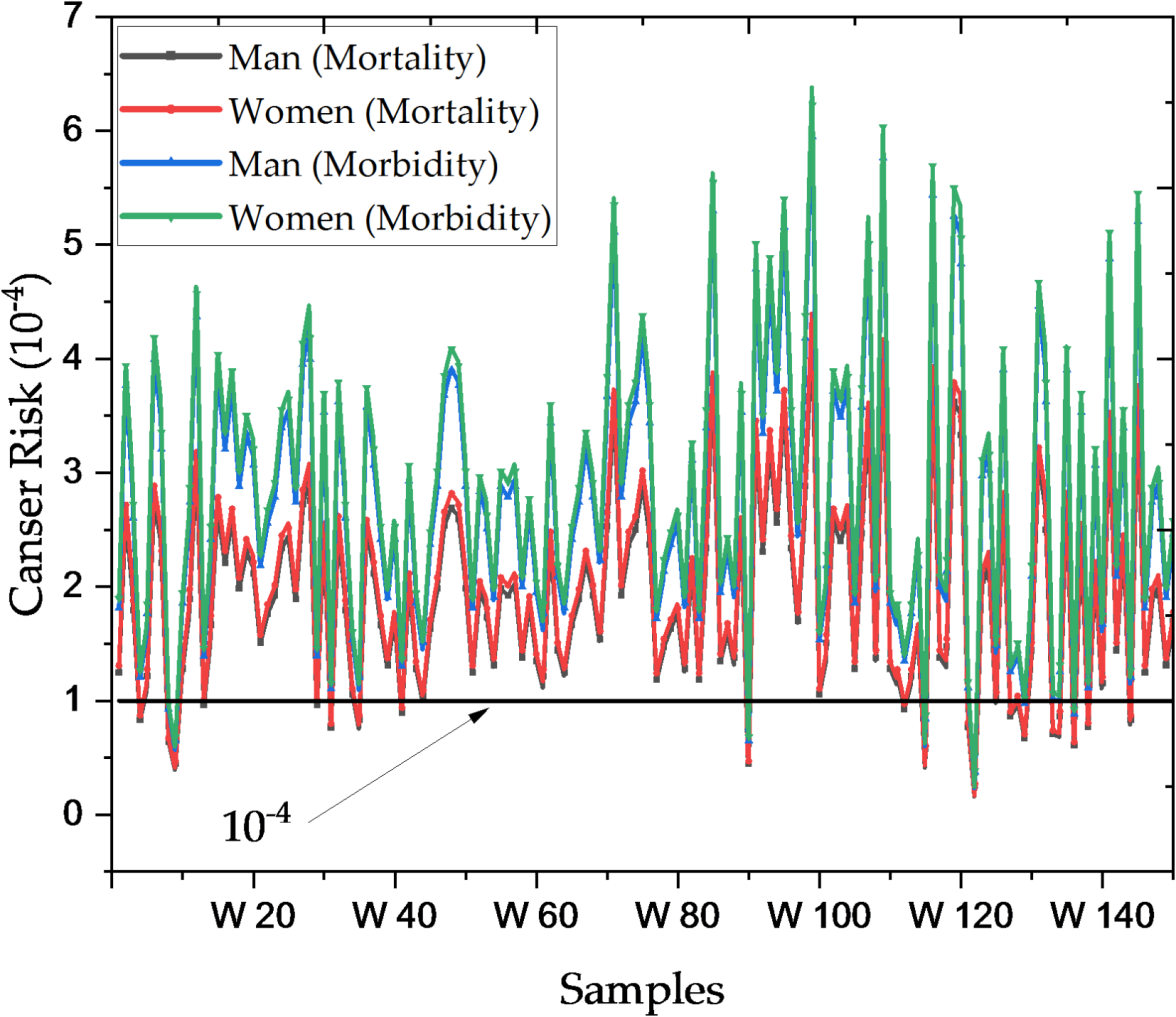
Cancer risk distribution for mortality and morbidity in Nasser Lake water samples.

CR was assessed based on Ra-226 concentrations, a primary contributor to radiation exposure^12^. Mortality CR for men ranges from 2.56 × 10^−5^ in W122 to 4.10 × 10^−4^ in W99, indicating an estimated 2.56 to 410 cancer-related deaths per 100,000 individuals exposed over a lifetime, while for women, mortality CR spans from 2.68 × 10^−5^ (W122) to 4.29 × 10^−4^ (W99), indicating slightly higher risks for women in some locations. These results suggest elevated localized radiation levels in specific areas of Nasser Lake, contributing to increased CR. For morbidity CR, which represents cancer incidence rather than mortality, men show values ranging from 3.72 × 10^−5^ (W122) to 5.95 × 10^−4^ (W99), while women exhibit a slightly higher range of 3.89 × 10^−5^ (W122) to 6.22 × 10^−4^ (W99), corresponding to an estimated 3.72 to 595 lifetime cancer cases per 100,000 individuals. Higher morbidity risks align with areas of elevated mortality risks, particularly in samples W109, W116, and W119, suggesting critical radiation hot spots. These hot spots may result from accumulated radioactive isotopes in sediments or local geological formations. Women exhibit slightly higher CR than men, likely due to their longer life expectancy, resulting in extended periods of low-dose radiation exposure. This highlights the importance of considering both biological and environmental factors^13^ when assessing health risks in Nasser Lake. The findings emphasize the urgent need for continuous monitoring programs to reduce radiation exposure, particularly in high-radiation areas. Preventive measures and public health strategies are essential to mitigate long-term risks. Figure 5 illustrates the distribution of CR for both mortality and morbidity across different water samples, highlighting specific hotspots with elevated radiation exposure.

### Water properties in relation to radioactive isotopes

pH and EC are essential parameters for assessing water quality. The pH scale, ranging from 0 to 14, indicates acidity or alkalinity of water, while EC measures the ability of water to conduct electricity based on ion concentration. These properties are influenced by factors such as pollutants, organic material, and dissolved compounds. In this study, a correlation was observed between elevated concentrations of NORs and higher EC values. However, this correlation does not necessarily imply a direct causal relationship, as EC is also affected by dissolved salts and other naturally occurring ions from geological sources. The pH values in the samples ranged from 6.23 to 7.9, with most samples falling between 6.5 and 7.5, suggesting relatively well-balanced water. Lower pH values, such as those recorded in samples W60 and W112, were associated with lower EC, indicating fewer dissolved ions. In contrast, higher pH values, as observed in samples W70 and W103, corresponded with increased EC, suggesting a greater presence of dissolved ions and enhanced conductivity. This pattern suggests a potential correlation between pH and EC, modulated by the presence of radioactive isotopes and other environmental variables.

Radiological activity varied significantly across the study area, primarily influenced by local geological formations and hydrological conditions. Additionally, it highlighted the increased vulnerability of children and infants to radiation exposure, emphasizing the need for specific protective measures tailored to these groups. CR evaluations identified radiation hotspots in Nasser Lake, necessitating continuous monitoring of these critical zones. To minimize risks for children and infants, additional screening of water sources intended for their consumption should be prioritized, with strict regulatory oversight to ensure compliance with safety standards. The observed relationship between water properties, pH, and EC further demonstrated the influence of radioactive isotopes on water’s chemical composition. This reinforces the importance of providing alternative low-radiation water sources for highly affected regions, particularly for infants and young children who are more susceptible to radiation exposure. These findings underscore the critical need for sustained monitoring to ensure water safty in regions with elevated NORs, thereby minimizing long-term health and environmental risks. Additionally, public awareness campaigns targeting families, schools, and healthcare providers should be implemented to educate communities on the risks of radiation exposure in drinking water and safe consumption practices. To mitigate radiological risks, potential strategies include water treatment techniques such as ion exchange and reverse osmosis, which are widely recognized for their efficacy in reducing radionuclide concentrations. For populations at higher risk, such as infants and young children, priority should be given to implementing these treatment methods in schools, daycare centers, and households in affected regions. Continuous surveillance in high-radiation zones is essential for maintaining safe drinking water standards and preventing chronic exposure-related health outcomes. Special monitoring protocols should be established for water sources primarily used by children, ensuring that exposure levels remain within internationally recognized safety thresholds.

## Conclusion

This study comprehensively assessed the radiological activity in Nasser Lake, focusing on naturally occurring radionuclides (NORs) including radium-226 (Ra-226), thorium-232 (Th-232), and potassium-40 (K-40). The findings revealed that although most water samples were within international safety limits, certain areas exhibited elevated radionuclide concentrations, posing potential risks, particularly for vulnerable populations such as children and infants. The assessment of annual effective doses (E_*ff*_) showed that adults’ exposure remained within safe limits, ranging from 15.5 to 266.15 micro sievert per year (µSv/y). However, children’s doses, ranging from 22.26 µSv/y to -362.92 µSv/y) exceeded the recommended public exposure limit of 100 µSv/y set by the international commission on radiological protection (ICRP), highlighting the need for targeted risk management. Cancer risk (CR) analysis identified specific radiation hotspots where elevated radionuclide levels increased both lifetime mortality and morbidity risks. The mortality risk for men ranged from 2.56 × 10^−5^ to 4.10 × 10^−4^, while for women, it ranged from 2.68 × 10^−5^ to 4.29 × 10^−4^, indicating an estimated probability of 2.56 to 410 cancer-related deathes per 100,000 individuals exposed over a lifetime. Morbidity risks followed a similar pattern, further reinforcing the need for continuous monitoring in high-radiation areas. The findings provide critical data that can inform national and international water quality regulations, aligning with established radiological safety standards. The study also explored the relationship between the acidity levels of water (pH), electrical conductivity (EC), and radionuclide concentrations. Samples with lower pH exhibited lower EC, whereas those with higher pH showed higher EC. This pattern suggests an association between ion levels and radionuclide activity, although further research is necessary to establish a direct causal relationship. In conclusion, while most of Nasser Lake’s water samples fall within acceptable safety limits, specific locations require further investigation. Continuous monitoring is crucial to ensure the protection of vulnerable populations, particularly children and infants. Implementing advanced water treatment methods, such as ion exchange and reverse osmosis, could help mitigate radionuclide concentrations in affected areas. Additionally, public awareness campaigns and regulatory measures should be considered to ensure long-term water safety. Effective mitigation strategies are essential for minimizing health risks associated with water consumption from Nasser Lake and ensuring compliance with international radiological safety standards.

## Electronic supplementary material

Below is the link to the electronic supplementary material.

Supplementary Material 1

## Data Availability

All data generated or analyzed during this study are included in the manuscript. Additional data or data files can be provided by the corresponding author upon request.
