# Supplementary material for: Radiological risks in Nasser lake water and their health and environmental implications
Source: Sci Rep. 2025 Apr 25;15:14545. doi: 10.1038/s41598-025-95257-7 (PMC12031930; doi:10.1038/s41598-025-95257-7)
Supplement: Supplementary file 1 — Supplementary Material 1 [file 41598_2025_95257_MOESM1_ESM.docx]

**Radiological Risks in Nasser Lake Water: Health Assessment and Environmental Impacts**

**Sub-elementary file**

Table S1: Radionuclide Activity, Effective Dose, Cancer Risk, and Water Properties in Nasser Lake Samples

| **Samples** | **Ra-226**  **(Bq/l)** | **Th-232**  **(Bq/l)** | **K-40**  **(Bq/l)** | **pH** | **EC**  **(µS/cm)** | **E*_ff_* (µSv/y)** | | | **CR (mortality)** | | **CR (morbidity)** | |
| --- | --- | --- | --- | --- | --- | --- | --- | --- | --- | --- | --- | --- |
|  |  |  |  |  |  | **Adults** | **Childs** | **Infants** | **Man** | **Women** | **Man** | **Women** |
| W 1 | 0.39±0.02 | 0.32±0.02 | 3.06±0.26 | 7.43 | 935 | 91.40 | 123.83 | 75.42 | 1.25 | 1.31 | 1.81 | 1.90 |
| W 2 | 0.81±0.04 | 0.34±0.02 | 15.35±1.32 | 7.42 | 917 | 152.50 | 217.42 | 134.73 | 2.59 | 2.72 | 3.76 | 3.94 |
| W 3 | 0.56±0.03 | 0.36±0.02 | 6.57±0.56 | 7.33 | 923 | 119.80 | 165.62 | 101.58 | 1.79 | 1.88 | 2.60 | 2.73 |
| W 4 | 0.26±0.01 | 0.36±0.02 | 2.68±0.23 | 7.39 | 914 | 77.80 | 100.52 | 60.18 | 0.83 | 0.87 | 1.21 | 1.27 |
| W 5 | 0.38±0.02 | 0.23±0.01 | 11.95±1.03 | 7.34 | 945 | 79.65 | 110.64 | 67.97 | 1.22 | 1.27 | 1.77 | 1.85 |
| W 6 | 0.86±0.04 | 0.18±0.01 | 8.30±0.71 | 6.9 | 936 | 141.10 | 208.67 | 130.83 | 2.75 | 2.89 | 4.00 | 4.19 |
| W 7 | 0.69±0.03 | 0.60±0.04 | 6.30±0.54 | 6.89 | 992 | 165.60 | 223.23 | 135.72 | 2.21 | 2.31 | 3.21 | 3.36 |
| W 8 | 0.20±0.011 | 0.19±0.01 | 2.02±0.17 | 6.85 | 953 | 49.85 | 66.68 | 40.43 | 0.64 | 0.67 | 0.93 | 0.97 |
| W 9 | 0.14±0.01 | 0.14±0.01 | 1.90±0.16 | 7.12 | 947 | 35.70 | 47.53 | 28.77 | 0.45 | 0.47 | 0.65 | 0.68 |
| W 10 | 0.4±0.02 | 0.25±0.02 | 2.44±0.21 | 6.87 | 963 | 84.75 | 117.43 | 72.08 | 1.28 | 1.34 | 1.86 | 1.95 |
| W 11 | 0.59±0.03 | 0.32±0.02 | 6.85±0.59 | 6.95 | 933 | 119.40 | 167.23 | 103.02 | 1.89 | 1.98 | 2.74 | 2.87 |
| W 12 | 0.94±0.05 | 0.58±0.03 | 1.35±0.11 | 6.58 | 912 | 198.30 | 275.03 | 168.87 | 3.01 | 3.15 | 4.37 | 4.57 |
| W 13 | 0.3±0.012 | 0.45±0.03 | 7.05±0.61 | 6.98 | 923 | 93.75 | 120.23 | 71.78 | 0.96 | 1.01 | 1.39 | 1.46 |
| W 14 | 0.52±0.03 | 0.35±0.02 | 14.22±1.22 | 7.11 | 934 | 113.05 | 155.72 | 95.39 | 1.67 | 1.74 | 2.42 | 2.53 |
| W 15 | 0.83±0.04 | 0.61±0.04 | 2.39±0.20 | 6.95 | 955 | 186.35 | 254.84 | 155.72 | 2.66 | 2.78 | 3.86 | 4.04 |
| W 16 | 0.69±0.03 | 0.35±0.02 | 7.62±0.65 | 7.02 | 967 | 136.85 | 192.61 | 118.85 | 2.21 | 2.31 | 3.21 | 3.36 |
| W 17 | 0.80±0.04 | 0.61±0.04 | 2.90±0.25 | 6.98 | 940 | 182.15 | 248.33 | 151.58 | 2.56 | 2.68 | 3.72 | 3.89 |
| W 18 | 0.62±0.03 | 0.46±0.03 | 9.04±0.78 | 6.97 | 932 | 139.70 | 190.89 | 116.61 | 1.99 | 2.08 | 2.88 | 3.02 |
| W 19 | 0.72±0.04 | 0.48±0.03 | 11.72±1.01 | 7.05 | 913 | 156.00 | 215.04 | 131.76 | 2.31 | 2.42 | 3.35 | 3.50 |
| W 20 | 0.66±0.03 | 0.18±0.01 | 13.49±1.16 | 7.23 | 924 | 113.10 | 165.27 | 103.23 | 2.11 | 2.21 | 3.07 | 3.21 |
| W 21 | 0.47±0.02 | 0.31±0.02 | 10.82±0.93 | 7.1 | 923 | 101.45 | 139.97 | 85.79 | 1.51 | 1.58 | 2.18 | 2.29 |
| W 22 | 0.55±0.03 | 0.53±0.03 | 7.85±0.67 | 6.98 | 902 | 137.95 | 184.28 | 111.68 | 1.76 | 1.85 | 2.56 | 2.68 |
| W 23 | 0.60±0.03 | 0.29±0.02 | 10.29±0.88 | 6.59 | 899 | 117.35 | 165.73 | 102.38 | 1.92 | 2.01 | 2.79 | 2.92 |
| W 24 | 0.73±0.04 | 0.48±0.03 | 12.33±1.06 | 6.58 | 905 | 157.40 | 217.21 | 133.14 | 2.34 | 2.45 | 3.39 | 3.55 |
| W 25 | 0.75±0.04 | 0.34±0.02 | 12.89±1.11 | 7.53 | 917 | 144.10 | 204.40 | 126.45 | 2.40 | 2.52 | 3.48 | 3.65 |
| W 26 | 0.59±0.03 | 0.25±0.01 | 12.56±1.08 | 6.68 | 1024 | 111.35 | 158.66 | 98.30 | 1.89 | 1.98 | 2.74 | 2.87 |
| W 27 | 0.85±0.04 | 0.60±0.04 | 7.49±0.64 | 6.9 | 903 | 188.00 | 257.95 | 157.80 | 2.72 | 2.85 | 3.95 | 4.14 |
| W 28 | 0.86±0.04 | 0.49±0.03 | 14.04±1.21 | 7.08 | 913 | 176.75 | 246.65 | 151.76 | 2.75 | 2.89 | 4.00 | 4.19 |
| W 29 | 0.30±0.15 | 0.35±0.02 | 11.70±1.01 | 7 | 923 | 82.25 | 107.98 | 65.03 | 0.96 | 1.01 | 1.39 | 1.46 |
| W 30 | 0.76±0.04 | 0.35±0.02 | 12.89±1.11 | 6.89 | 903 | 146.65 | 207.80 | 128.51 | 2.43 | 2.55 | 3.53 | 3.70 |
| W 31 | 0.24±0.01 | 0.28±0.02 | 8.38±0.72 | 6.66 | 912 | 65.85 | 86.28 | 51.93 | 0.76 | 0.80 | 1.10 | 1.16 |
| W 32 | 0.78±0.04 | 0.46±0.03 | 7.53±0.65 | 6.89 | 923 | 162.10 | 225.61 | 138.69 | 2.50 | 2.62 | 3.62 | 3.80 |
| W 33 | 0.56±0.03 | 0.40±0.02 | 10.81±0.93 | 7.02 | 908 | 124.40 | 170.52 | 104.28 | 1.79 | 1.88 | 2.60 | 2.73 |
| W 34 | 0.33±0.02 | 0.31±0.02 | 11.70±1.01 | 6.98 | 923 | 81.85 | 109.59 | 66.47 | 1.06 | 1.11 | 1.53 | 1.61 |
| W 35 | 0.26±0.01 | 0.31±0.02 | 11.50±0.99 | 6.69 | 923 | 72.05 | 94.40 | 56.81 | 0.83 | 0.87 | 1.21 | 1.27 |
| W 36 | 0.77±0.04 | 0.46±0.03 | 7.53±0.65 | 7.05 | 915 | 160.70 | 223.44 | 137.31 | 2.47 | 2.58 | 3.58 | 3.75 |
| W 37 | 0.66±0.03 | 0.31±0.02 | 9.50±0.82 | 7.18 | 908 | 128.05 | 181.20 | 112.01 | 2.11 | 2.21 | 3.07 | 3.21 |
| W 38 | 0.52±0.03 | 0.37±0.02 | 11.79±1.01 | 6.9 | 917 | 115.35 | 158.17 | 96.74 | 1.67 | 1.74 | 2.42 | 2.53 |
| W 39 | 0.41±0.02 | 0.25±0.01 | 12.07±1.04 | 6.8 | 925 | 86.15 | 119.60 | 73.46 | 1.31 | 1.38 | 1.90 | 2.00 |
| W 40 | 0.52±0.03 | 0.32±0.02 | 14.72±1.27 | 6.89 | 945 | 109.60 | 152.04 | 93.36 | 1.67 | 1.74 | 2.42 | 2.53 |
| W 41 | 0.28±0.01 | 0.21±0.01 | 10.78±0.93 | 7.15 | 972 | 63.35 | 86.49 | 52.82 | 0.90 | 0.94 | 1.30 | 1.36 |
| W 42 | 0.63±0.03 | 0.17±0.01 | 10.21±0.88 | 7.25 | 994 | 107.75 | 157.54 | 98.42 | 2.02 | 2.11 | 2.93 | 3.07 |
| W 43 | 0.40±0.02 | 0.31±0.02 | 10.17±0.88 | 7.14 | 943 | 91.65 | 124.78 | 76.13 | 1.28 | 1.34 | 1.86 | 1.95 |
| W 44 | 0.33±0.02 | 0.26±0.02 | 11.32±0.97 | 7.36 | 987 | 75.82 | 102.86 | 62.68 | 1.04 | 1.09 | 1.51 | 1.58 |
| W 45 | 0.51±0.02 | 0.40±0.02 | 8.61±0.74 | 6.79 | 1021 | 117.40 | 159.67 | 97.38 | 1.63 | 1.71 | 2.37 | 2.48 |
| W 46 | 0.62±0.03 | 0.15±0.01 | 9.62±0.83 | 6.79 | 1002 | 104.05 | 152.92 | 95.69 | 1.99 | 2.08 | 2.88 | 3.02 |
| W 47 | 0.79±0.04 | 0.34±0.02 | 15.71±1.35 | 7.06 | 1043 | 150.05 | 213.55 | 132.26 | 2.54 | 2.66 | 3.68 | 3.85 |
| W 48 | 0.84±0.04 | 0.54±0.03 | 9.20±0.79 | 6.59 | 997 | 179.70 | 248.43 | 152.37 | 2.69 | 2.82 | 3.90 | 4.09 |
| W 49 | 0.81±0.04 | 0.34±0.02 | 15.32±1.32 | 6.98 | 983 | 152.50 | 217.42 | 134.73 | 2.59 | 2.72 | 3.76 | 3.94 |
| W 50 | 0.62±0.03 | 0.15±0.01 | 10.71±0.92 | 6.78 | 995 | 104.05 | 152.92 | 95.69 | 1.99 | 2.08 | 2.88 | 3.02 |
| W 51 | 0.39±0.02 | 0.26±0.02 | 15.02±1.29 | 6.59 | 1025 | 84.50 | 116.48 | 71.37 | 1.25 | 1.31 | 1.81 | 1.90 |
| W 52 | 0.61±0.03 | 0.15±0.01 | 8.88±0.76 | 7.02 | 1258 | 102.65 | 150.75 | 94.31 | 1.95 | 2.05 | 2.83 | 2.97 |
| W 53 | 0.54±0.03 | 0.54±0.03 | 11.37±0.98 | 7.51 | 993 | 137.70 | 183.33 | 110.97 | 1.73 | 1.81 | 2.51 | 2.63 |
| W 54 | 0.41±0.02 | 0.10±0.01 | 12.31±1.03 | 6.9 | 982 | 68.90 | 101.22 | 63.33 | 1.31 | 1.38 | 1.90 | 2.00 |
| W 55 | 0.62±0.03 | 0.15±0.01 | 9.62±0.83 | 7.2 | 890 | 104.05 | 152.92 | 95.69 | 1.99 | 2.08 | 2.88 | 3.02 |
| W 56 | 0.60±0.03 | 0.09±0.01 | 9.20±0.79 | 6.69 | 878 | 94.35 | 141.23 | 88.88 | 1.92 | 2.01 | 2.79 | 2.92 |
| W 57 | 0.62±0.03 | 0.14±0.01 | 13.96±1.20 | 6.89 | 901 | 102.90 | 151.69 | 95.01 | 1.99 | 2.08 | 2.88 | 3.02 |
| W 58 | 0.43±0.02 | 0.39±0.03 | 12.31±1.06 | 6.78 | 898 | 105.05 | 141.09 | 85.67 | 1.38 | 1.44 | 2.00 | 2.09 |
| W 59 | 0.57±0.03 | 0.42±0.02 | 11.55±0.99 | 7.25 | 1002 | 128.10 | 175.14 | 107.01 | 1.83 | 1.91 | 2.65 | 2.77 |
| W 60 | 0.42±0.02 | 0.37±0.02 | 11.81±1.02 | 6.23 | 897 | 101.35 | 136.47 | 82.94 | 1.35 | 1.41 | 1.95 | 2.04 |
| W 61 | 0.38±0.02 | 0.25±0.02 | 11.11±0.96 | 7.38 | 1254 | 81.95 | 113.09 | 69.32 | 1.22 | 1.27 | 1.77 | 1.85 |
| W 62 | 0.74±0.04 | 0.38±0.02 | 10.09±0.87 | 6.89 | 892 | 147.30 | 207.13 | 127.77 | 2.37 | 2.48 | 3.44 | 3.60 |
| W 63 | 0.45±0.02 | 0.31±0.02 | 12.36±1.06 | 6.72 | 901 | 98.65 | 135.63 | 83.03 | 1.44 | 1.51 | 2.09 | 2.19 |
| W 64 | 0.39±0.02 | 0.27±0.02 | 11.33±0.97 | 7.08 | 918 | 85.65 | 117.71 | 72.05 | 1.25 | 1.31 | 1.81 | 1.90 |
| W 65 | 0.52±0.02 | 0.35±0.02 | 9.66±0.83 | 7.28 | 1025 | 113.05 | 155.72 | 95.39 | 1.67 | 1.74 | 2.42 | 2.53 |
| W 66 | 0.59±0.03 | 0.36±0.02 | 12.06±1.04 | 6.93 | 892 | 124.00 | 172.13 | 105.72 | 1.89 | 1.98 | 2.74 | 2.87 |
| W 67 | 0.69±0.03 | 0.24±0.01 | 13.38±1.15 | 6.86 | 925 | 124.20 | 179.13 | 111.42 | 2.21 | 2.31 | 3.21 | 3.36 |
| W 68 | 0.60±0.03 | 0.12±0.01 | 12.27±1.06 | 6.58 | 893 | 97.80 | 144.90 | 90.90 | 1.92 | 2.01 | 2.79 | 2.92 |
| W 69 | 0.48±0.02 | 0.26±0.02 | 10.65±0.92 | 7.8 | 1203 | 97.10 | 136.01 | 83.79 | 1.54 | 1.61 | 2.23 | 2.34 |
| W 70 | 0.79±0.04 | 0.61±0.05 | 9.35±0.80 | 7.29 | 1025 | 180.75 | 246.16 | 150.20 | 2.53 | 2.65 | 3.67 | 3.84 |
| W 71 | 1.1±0.05 | 0.39±0.02 | 13.53±1.16 | 7.38 | 1258 | 198.85 | 286.48 | 178.13 | 3.52 | 3.69 | 5.11 | 5.35 |
| W 72 | 0.60±0.03 | 0.37±0.02 | 10.83±0.93 | 7.51 | 1364 | 126.55 | 175.53 | 107.78 | 1.92 | 2.01 | 2.79 | 2.92 |
| W 73 | 0.74±0.04 | 0.50±0.03 | 10.60±0.91 | 6.98 | 895 | 161.10 | 221.83 | 135.87 | 2.37 | 2.48 | 3.44 | 3.60 |
| W 74 | 0.78±0.04 | 0.29±0.02 | 12.55±1.08 | 6.93 | 879 | 142.55 | 204.79 | 127.22 | 2.50 | 2.62 | 3.62 | 3.80 |
| W 75 | 0.90±0.04 | 0.13±0.01 | 13.28±1.14 | 6.79 | 901 | 140.95 | 211.23 | 132.98 | 2.88 | 3.02 | 4.18 | 4.38 |
| W 76 | 0.74±0.03 | 0.54±0.03 | 10.90±0.94 | 6.98 | 982 | 165.70 | 226.73 | 138.57 | 2.37 | 2.48 | 3.44 | 3.60 |
| W 77 | 0.37±0.02 | 0.57±0.03 | 10.07±0.87 | 7.8 | 1245 | 117.35 | 150.12 | 89.54 | 1.19 | 1.24 | 1.72 | 1.80 |
| W 78 | 0.46±0.02 | 0.24±0.01 | 10.28±0.88 | 6.89 | 983 | 92.00 | 129.22 | 79.68 | 1.47 | 1.54 | 2.14 | 2.24 |
| W 79 | 0.51±0.02 | 0.43±0.03 | 10.80±0.93 | 6.9 | 897 | 120.85 | 163.35 | 99.41 | 1.63 | 1.71 | 2.37 | 2.48 |
| W 80 | 0.54±0.03 | 0.44±0.03 | 11.74±1.01 | 6.78 | 968 | 126.20 | 171.08 | 104.22 | 1.73 | 1.81 | 2.51 | 2.63 |
| W 81 | 0.40±0.02 | 0.50±0.03 | 5.16±0.44 | 6.85 | 897 | 113.50 | 148.05 | 88.95 | 1.28 | 1.34 | 1.86 | 1.95 |
| W 82 | 0.67±0.03 | 0.33±0.02 | 15.53±1.16 | 6.96 | 876 | 131.75 | 185.82 | 114.74 | 2.15 | 2.25 | 3.11 | 3.26 |
| W 83 | 0.37±0.02 | 0.57±0.03 | 10.07±0.87 | 6.68 | 928 | 117.35 | 150.12 | 89.54 | 1.19 | 1.24 | 1.72 | 1.80 |
| W 84 | 0.73±0.04 | 0.34±0.02 | 11.88±1.02 | 6.95 | 983 | 141.30 | 200.06 | 123.69 | 2.34 | 2.45 | 3.39 | 3.55 |
| W 85 | 1.14±0.06 | 0.29±0.02 | 16.57±1.43 | 6.91 | 839 | 192.95 | 282.91 | 176.90 | 3.65 | 3.82 | 5.30 | 5.55 |
| W 86 | 0.42±0.02 | 0.36±0.02 | 12.48±1.07 | 6.8 | 869 | 100.20 | 135.24 | 82.26 | 1.35 | 1.41 | 1.95 | 2.04 |
| W 87 | 0.50±0.02 | 0.39±0.02 | 13.53±1.16 | 7.08 | 1038 | 114.85 | 156.28 | 95.33 | 1.60 | 1.68 | 2.32 | 2.43 |
| W 88 | 0.43±0.02 | 0.28±0.02 | 10.47±0.90 | 7.9 | 1452 | 92.40 | 127.61 | 78.24 | 1.38 | 1.44 | 2.00 | 2.09 |
| W 89 | 0.76±0.04 | 0.46±0.03 | 11.71±1.01 | 6.89 | 983 | 159.30 | 221.27 | 135.93 | 2.43 | 2.55 | 3.53 | 3.70 |
| W 90 | 0.14±0.01 | 0.27±0.02 | 7.05±0.61 | 6.58 | 869 | 50.65 | 63.46 | 37.55 | 0.45 | 0.47 | 0.65 | 0.68 |
| W 91 | 1.03±0.05 | 0.83±0.05 | 8.70±0.75 | 7.28 | 1368 | 239.65 | 325.19 | 198.17 | 3.30 | 3.46 | 4.79 | 5.01 |
| W 92 | 0.72±0.04 | 0.56±0.03 | 11.32±0.97 | 7.35 | 1458 | 165.20 | 224.84 | 137.16 | 2.31 | 2.42 | 3.35 | 3.50 |
| W 93 | 1.00±0.05 | 0.82±0.05 | 9.10±0.78 | 7.02 | 1025 | 234.94 | 318.44 | 193.98 | 3.22 | 3.37 | 4.67 | 4.89 |
| W 94 | 0.80±0.04 | 0.20±0.01 | 13.72±1.18 | 7.19 | 983 | 135.00 | 198.10 | 123.90 | 2.56 | 2.68 | 3.72 | 3.89 |
| W 95 | 1.11±0.06 | 0.39±0.02 | 7.83±0.67 | 6.59 | 896 | 200.25 | 288.65 | 179.51 | 3.56 | 3.72 | 5.16 | 5.40 |
| W 96 | 0.73±0.04 | 0.55±0.03 | 11.32±0.97 | 6.9 | 897 | 165.45 | 225.79 | 137.87 | 2.34 | 2.45 | 3.39 | 3.55 |
| W 97 | 0.53±0.03 | 0.49±0.03 | 11.86±1.02 | 6.89 | 908 | 130.55 | 175.04 | 106.22 | 1.70 | 1.78 | 2.46 | 2.58 |
| W 98 | 0.90±0.04 | 0.63±0.04 | 13.02±1.12 | 6.78 | 924 | 198.45 | 272.48 | 166.73 | 2.88 | 3.02 | 4.18 | 4.38 |
| W 99 | 1.28±0.06 | 0.37±0.02 | 15.19±1.31 | 7.02 | 983 | 221.75 | 323.09 | 201.62 | 4.10 | 4.29 | 5.95 | 6.23 |
| W 100 | 0.33±0.02 | 0.25±0.02 | 4.94±0.43 | 6.93 | 839 | 74.95 | 102.24 | 62.42 | 1.06 | 1.11 | 1.53 | 1.61 |
| W 101 | 0.47±0.02 | 0.52±0.03 | 5.58±0.48 | 7.08 | 852 | 125.60 | 165.69 | 99.96 | 1.51 | 1.58 | 2.18 | 2.29 |
| W 102 | 0.80±0.04 | 0.20±0.01 | 13.72±1.18 | 6.8 | 790 | 135.00 | 198.10 | 123.90 | 2.56 | 2.68 | 3.72 | 3.89 |
| W 103 | 0.75±0.04 | 0.35±0.02 | 2.04±0.18 | 7.51 | 1456 | 145.25 | 205.63 | 127.13 | 2.40 | 2.52 | 3.48 | 3.65 |
| W 104 | 0.79±0.04 | 0.43±0.03 | 2.05±0.18 | 6.9 | 892 | 160.05 | 224.11 | 138.05 | 2.53 | 2.65 | 3.67 | 3.84 |
| W 105 | 0.40±0.02 | 0.19±0.01 | 6.21±0.53 | 6.49 | 789 | 77.85 | 110.08 | 68.03 | 1.28 | 1.34 | 1.86 | 1.95 |
| W 106 | 0.77±0.04 | 0.18±0.01 | 3.96±0.34 | 6.59 | 892 | 128.50 | 189.14 | 118.41 | 2.47 | 2.58 | 3.58 | 3.75 |
| W 107 | 1.03±0.05 | 0.61±0.04 | 4.74±0.41 | 6.48 | 898 | 214.35 | 298.24 | 183.32 | 3.30 | 3.46 | 4.79 | 5.01 |
| W 108 | 0.43±0.02 | 0.16±0.01 | 3.64±0.31 | 6.83 | 869 | 78.60 | 112.91 | 70.14 | 1.38 | 1.44 | 2.00 | 2.09 |
| W 109 | 1.24±0.06 | 0.09±0.01 | 9.54±0.82 | 7.08 | 1002 | 183.95 | 280.11 | 177.20 | 3.97 | 4.16 | 5.76 | 6.03 |
| W 110 | 0.40±0.02 | 0.32±0.02 | 2.57±0.22 | 7.8 | 1736 | 92.80 | 126.00 | 76.80 | 1.28 | 1.34 | 1.86 | 1.95 |
| W 111 | 0.38±0.02 | 0.22±0.01 | 3.48±0.30 | 6.72 | 982 | 78.50 | 109.41 | 67.29 | 1.22 | 1.27 | 1.77 | 1.85 |
| W 112 | 0.29±0.02 | 0.33±0.03 | 2.10±0.18 | 6.84 | 689 | 78.55 | 103.36 | 62.30 | 0.93 | 0.97 | 1.35 | 1.41 |
| W 113 | 0.38±0.02 | 0.12±0.01 | 2.86±0.25 | 6.95 | 892 | 67.00 | 97.16 | 60.54 | 1.22 | 1.27 | 1.77 | 1.85 |
| W 114 | 0.47±0.02 | 0.52±0.03 | 5.58±0.48 | 7.05 | 1025 | 125.60 | 165.69 | 99.96 | 1.51 | 1.58 | 2.18 | 2.29 |
| W 115 | 0.18±0.01 | 0.13±0.01 | 4.33±0.37 | 7.61 | 1702 | 40.15 | 54.99 | 33.62 | 0.58 | 0.60 | 0.84 | 0.88 |
| W 116 | 1.17±0.06 | 0.89±0.05 | 4.48±0.38 | 6.8 | 896 | 266.15 | 362.92 | 221.54 | 3.75 | 3.93 | 5.44 | 5.69 |
| W 117 | 0.43±0.02 | 0.41±0.03 | 4.49±0.39 | 7.08 | 987 | 107.35 | 143.54 | 87.02 | 1.38 | 1.44 | 2.00 | 2.09 |
| W 118 | 0.46±0.02 | 0.20±0.01 | 8.67±0.75 | 7 | 890 | 87.40 | 124.32 | 76.98 | 1.47 | 1.54 | 2.14 | 2.24 |
| W 119 | 1.13±0.06 | 0.72±0.04 | 5.27±0.45 | 6.89 | 792 | 241.00 | 333.41 | 204.54 | 3.62 | 3.79 | 5.25 | 5.50 |
| W 120 | 1.04±0.05 | 0.96±0.06 | 4.21±0.36 | 6.5 | 893 | 256.00 | 343.28 | 208.32 | 3.33 | 3.49 | 4.83 | 5.06 |
| W 121 | 0.24±0.01 | 0.13±0.01 | 2.49±0.21 | 6.87 | 986 | 48.55 | 68.01 | 41.90 | 0.77 | 0.81 | 1.12 | 1.17 |
| W 122 | 0.08±0.003 | 0.04±0.01 | 3.60±0.31 | 6.97 | 897 | 15.80 | 22.26 | 13.74 | 0.26 | 0.27 | 0.37 | 0.39 |
| W 123 | 0.64±0.03 | 0.27±0.02 | 2.36±0.20 | 7.9 | 1586 | 120.65 | 171.96 | 106.55 | 2.05 | 2.15 | 2.97 | 3.11 |
| W 124 | 0.65±0.03 | 0.42±0.03 | 2.58±0.22 | 7.5 | 1458 | 139.30 | 192.50 | 118.05 | 2.08 | 2.18 | 3.02 | 3.16 |
| W 125 | 0.32±0.02 | 0.27±0.02 | 4.46±0.38 | 7.26 | 1546 | 75.85 | 102.52 | 62.39 | 1.03 | 1.07 | 1.49 | 1.56 |
| W 126 | 0.84±0.04 | 0.20±0.01 | 2.98±0.26 | 7.08 | 1005 | 140.60 | 206.78 | 129.42 | 2.69 | 2.82 | 3.90 | 4.09 |
| W 127 | 0.27±0.01 | 0.36±0.02 | 4.30±0.37 | 6.93 | 1365 | 79.20 | 102.69 | 61.56 | 0.86 | 0.91 | 1.25 | 1.31 |
| W 128 | 0.31±0.02 | 0.33±0.02 | 4.22±0.36 | 7.18 | 1289 | 81.35 | 107.70 | 65.06 | 0.99 | 1.04 | 1.44 | 1.51 |
| W 129 | 0.21±0.01 | 0.37±0.02 | 5.16±0.44 | 7.28 | 1863 | 71.95 | 90.90 | 53.96 | 0.67 | 0.70 | 0.98 | 1.02 |
| W 130 | 0.45±0.02 | 0.31±0.02 | 4.27±0.37 | 6.9 | 986 | 98.65 | 135.63 | 83.03 | 1.44 | 1.51 | 2.09 | 2.19 |
| W 131 | 0.96±0.05 | 0.48±0.03 | 2.24±0.19 | 6.59 | 893 | 189.60 | 267.12 | 164.88 | 3.08 | 3.22 | 4.46 | 4.67 |
| W 132 | 0.78±0.04 | 0.47±0.03 | 2.09±0.18 | 6.38 | 980 | 163.25 | 226.84 | 139.37 | 2.50 | 2.62 | 3.62 | 3.80 |
| W 133 | 0.22±0.01 | 0.25±0.02 | 3.91±0.34 | 6.81 | 897 | 59.55 | 78.37 | 47.24 | 0.70 | 0.74 | 1.02 | 1.07 |
| W 134 | 0.27±0.01 | 0.36±0.02 | 4.30±0.37 | 7.08 | 987 | 79.20 | 102.69 | 61.56 | 0.86 | 0.91 | 1.25 | 1.31 |
| W 135 | 0.84±0.04 | 0.19±0.01 | 2.98±0.26 | 7.4 | 1325 | 139.45 | 205.56 | 128.75 | 2.69 | 2.82 | 3.90 | 4.09 |
| W 136 | 0.19±0.01 | 0.18±0.02 | 3.42±0.29 | 6.89 | 968 | 47.30 | 63.28 | 38.37 | 0.61 | 0.64 | 0.88 | 0.92 |
| W 137 | 0.76±0.04 | 0.22±0.01 | 2.78±0.24 | 6.29 | 963 | 131.70 | 191.87 | 119.73 | 2.43 | 2.55 | 3.53 | 3.70 |
| W 138 | 0.24±0.01 | 0.36±0.02 | 2.65±0.23 | 6.51 | 897 | 75.00 | 96.18 | 57.42 | 0.77 | 0.81 | 1.12 | 1.17 |
| W 139 | 0.66±0.03 | 0.24±0.02 | 3.88±0.33 | 6.39 | 869 | 120.00 | 172.62 | 107.28 | 2.11 | 2.21 | 3.07 | 3.21 |
| W 140 | 0.36±0.02 | 0.15±0.01 | 5.23±0.45 | 6.8 | 798 | 67.65 | 96.50 | 59.81 | 1.15 | 1.21 | 1.67 | 1.75 |
| W 141 | 1.05±0.05 | 0.29±0.02 | 10.59±0.91 | 6.84 | 569 | 180.35 | 263.38 | 164.48 | 3.36 | 3.52 | 4.88 | 5.11 |
| W 142 | 0.45±0.02 | 0.21±0.02 | 2.80±0.24 | 6.79 | 986 | 87.15 | 123.38 | 76.28 | 1.44 | 1.51 | 2.09 | 2.19 |
| W 143 | 0.73±0.04 | 0.65±0.04 | 11.06±0.95 | 6.86 | 698 | 176.95 | 238.04 | 144.62 | 2.34 | 2.45 | 3.39 | 3.55 |
| W 144 | 0.26±0.01 | 0.31±0.02 | 3.76±0.32 | 7.8 | 1582 | 72.05 | 94.40 | 56.81 | 0.83 | 0.87 | 1.21 | 1.27 |
| W 145 | 1.12±0.06 | 0.62±0.04 | 3.34±0.29 | 6.9 | 968 | 228.10 | 318.99 | 196.41 | 3.59 | 3.76 | 5.20 | 5.45 |
| W 146 | 0.39±0.02 | 0.26±0.02 | 15.02±1.29 | 7.09 | 897 | 84.50 | 116.48 | 71.37 | 1.25 | 1.31 | 1.81 | 1.90 |
| W 147 | 0.59±0.03 | 0.35±0.02 | 13.50±1.16 | 7.19 | 1265 | 122.85 | 170.91 | 105.05 | 1.89 | 1.98 | 2.74 | 2.87 |
| W 148 | 0.60±0.03 | 0.57±0.03 | 9.54±0.82 | 7.28 | 1325 | 149.55 | 200.03 | 121.28 | 1.92 | 2.01 | 2.79 | 2.92 |
| W 149 | 0.41±0.02 | 0.47±0.03 | 15.67±1.35 | 7.62 | 1245 | 111.45 | 146.55 | 88.31 | 1.31 | 1.38 | 1.90 | 2.00 |
| W 150 | 0.53±0.03 | 0.26±0.02 | 10.30±0.89 | 6.79 | 968 | 104.10 | 146.86 | 90.69 | 1.70 | 1.78 | 2.46 | 2.58 |
| **Minimum** | **0.08±0.003** | **0.04±0.01** | **1.35±0.11** | **6.23** | **569** | **15.8** | **22.26** | **13.74** | **0.257** | **0.27** | **0.37** | **0.39** |
| **Maximum** | **1.28±0.06** | **0.96±0.06** | **16.57±1.43** | **7.9** | **1863** | **266.15** | **362.92** | **221.535** | **4.1** | **4.29** | **5.95** | **6.23** |
| **Mean** | **0.59±0.03** | **0.36±0.05** | **8.6±0.75** | **6.99** | **998.43** | **123.7** | **171.9** | **105.6** | **1.88** | **1.98** | **2.74** | **2.87** |

Table S2 Calculated values of the lower confidence limit (LCL), upper confidence limit (UCL), detection limit (Dl) and minimum detectable activity (MDA) (Bq/l) for the NaI (Tl) Scintillation detector

| **Energy (keV)** | **Radionuclide** | **LCL** | **UCL** | **Dl** | **MDA** |
| --- | --- | --- | --- | --- | --- |
| 609.3 | ^214^Bi | 0.3837 | 0.4853 | 0.051 | 0.074 |
| 1120.2 | ^214^Bi | 0.3561 | 0.6019 | 0.163 | 0.237 |
| 1764.5 | ^214^Bi | 0.2409 | 0.6040 | 0.274 | 0.399 |
| 238.6 | ^212^Pb | 1.0125 | 1.2166 | 0.062 | 0.093 |
| 911.2 | ^228^Ac | 0.2034 | 0.2695 | 0.031 | 0.035 |
| 1460.8 | ^40^K | 3.1107 | 5.0680 | 1.336 | 1.942 |
